# Vincristine Treatment Protects Against Podocyte Damage in Focal Segmental Glomerulosclerosis

**DOI:** 10.1016/j.ekir.2025.08.002

**Published:** 2025-08-14

**Authors:** William J. Mason, Jennifer C. Chandler, Alice M. Gage, Gideon Pomeranz, Karen L. Price, Marilina Antonelou, Scott R. Henderson, Laura Perin, Stefano Da Sacco, Alan D. Salama, David A. Long, Ruth J. Pepper

**Affiliations:** 1 Developmental Biology and Cancer Research and Teaching Department, Great Ormond Street Institute of Child Health, Faculty of Population Health Sciences, University College London, London, UK; 2 University College of London Centre for Kidney and Bladder Health, Division of Medicine, Faculty of Medical Sciences, University College London, London, UK; 3 Royal Free Hospital, University College London Medical School, Faculty of Medical Sciences, University College London, London, UK; 4 GOFARR Laboratory for Organ Regenerative Research and Cell Therapeutics in Urology, Division of Urology, The Saban Research Institute, Children’s Hospital Los Angeles, Los Angeles, California, USA; 5 Keck School of Medicine, University of Southern California, Los Angeles, California, USA

**Keywords:** F-actin, focal segmental glomerulosclerosis, glomerular disease, microtubules, podocytes, vincristine

## Abstract

**Introduction:**

Focal segmental glomerulosclerosis (FSGS) is associated with podocyte damage resulting in cytoskeletal alterations leading to foot process effacement. Vincristine is a chemoprotective drug which alters cytoskeletal microtubules and has been used clinically to reverse FSGS. However, the mechanisms underlying the beneficial effect of vincristine are not understood.

**Methods:**

Immortalized human podocytes were exposed to serum obtained from an adult index patient with FSGS before, during, and after vincristine treatment. We examined the podocyte transcriptome by RNA-sequencing alongside cytoskeletal structure and filtration barrier integrity using a glomerulus-on-a-chip (GOAC) model.

**Results:**

Podocytes exposed to serum from the index patient with FSGS during or after vincristine treatment contained lower levels of genes associated with microtubule function compared with cells stimulated with serum collected during disease presentation. Presentation serum altered tubulin and F-actin patterning, changes prevented when podocytes were exposed to sera taken during or after vincristine treatment and when vincristine was added to presentation serum. IgG depletion experiments revealed that podocyte damage initiated by the index patient presentation serum was not due to circulating autoantibodies. Addition of serum from 3 more patients with FSGS also caused podocyte tubulin disorganization which was prevented by vincristine. Addition of FSGS serum to the GOAC led to increased albumin permeability in 2 patients, which could be prevented by vincristine.

**Conclusion:**

Vincristine protects against pathological changes induced by FSGS serum, with preservation of tubulin and F-actin organization in podocytes. Understanding whether vincristine exerts similar effects in other patients with FSGS warrants further investigation to advance our knowledge of this alternative therapeutic.

## Graphical abstract

**Figure:**
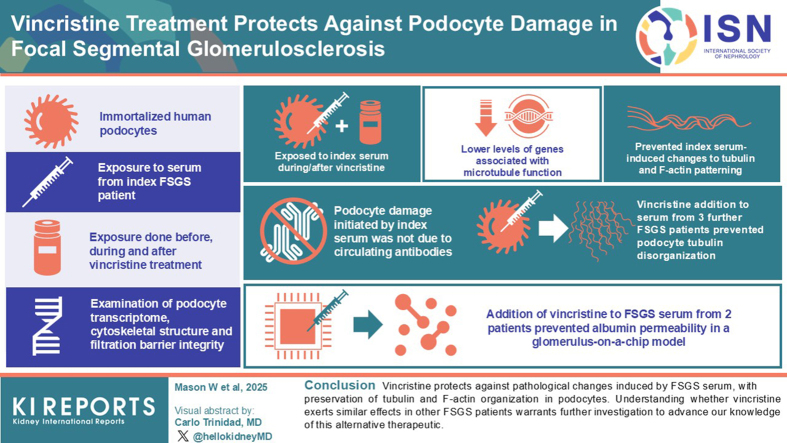

See Commentary on Page 3727

Chronic kidney disease affects approximately 10% of the global population.^1^ FSGS is associated with chronic kidney disease, leading to proteinuria and ultimately progression to end-stage kidney disease.^2^ Treatment strategies such as steroids and immunosuppression are ineffective in some patients with FSGS.^3^ Therefore, identifying disease mechanisms which underlie FSGS and developing new therapies to target them is needed.

FSGS is associated with damage to glomerular podocyte epithelial cells.^2^^,^^3^ This can be triggered by podocyte gene mutations,^4^ circulating factors,^5^ or autoantibodies to slit diaphragm molecules such as nephrin^6^^,^^7^; however, in some cases, the cause remains elusive. Podocytes have a unique highly branched architecture critical for maintaining filtration barrier integrity. This specialized shape relies on the cytoskeleton comprised of microtubules and intermediate filaments, located in the cell body and primary processes as well as F-actin, located predominately in the foot processes.^8^ The F-actin and microtubule networks are intricately interlinked, through synergistic regulation of dynamics,^9^ regulation of cell shape, migration and stiffness,^9^^,^^10^ mitotic spindle positioning,^11^ and are controlled by identical proteins, such as profilin^12^ and formin-2.^13^ Disruption to cytoskeletal architecture occurs in preclinical *in vivo*^14^ and *in vitro*^15^^,^^16^ FSGS models, as well as in cultured podocytes exposed to serum of patients with FSGS.^17^, ^18^, ^19^ This reorganization of the cytoskeleton changes podocyte shape resulting in cell detachment and foot process effacement.^8^^,^^20^

Several studies have tested whether therapies which preserve cytoskeletal structure improve FSGS. A small molecule targeting the GTPase dynamin, which interacts with actin, promoted stress fiber formation in cultured podocytes^21^ and improved disease progression in preclinical chronic kidney disease models.^22^ Systemic overexpression of the G-actin sequestering peptide, thymosin β-4, protected the podocyte cytoskeleton in an FSGS model of adriamycin nephropathy,^15^ through F-actin preservation. Vincristine, a chemotherapy drug^23^ which prevents microtubule polymerization resulting in cell cycle arrest,^24^ caused remission in some patients with steroid-resistant FSGS.^25^^,^^26^ These clinical findings show that vincristine is a promising therapeutic strategy for some FSGS cases. However, the mechanisms underlying the protective effect of vincristine are not fully understood. This study aimed to address this knowledge gap by collecting serum before, during, and after vincristine treatment from a patient with steroid resistant FSGS. We assessed the impact of vincristine therapy on podocyte structure and transcriptomics alongside glomerular filtration barrier integrity using human cellular models.

## Methods

### Patient Samples

Our index patient (Table 1**,** patient 1) was known to pediatric services^25^ with biopsy-proven FSGS. They subsequently presented to the adult renal clinic with a relapse of nephrotic syndrome, where genetic analysis using a nephrotic panel was performed followed by enrolment into The UK 100,000 Genome Project.^27^ Serum was collected at the following times: (i) before treatment when they presented with disease, (ii) during vincristine treatment, and (iii) during remission while off vincristine treatment (Table 2). We also collected samples (Table 2, patients 2–4) from 3 more patients with steroid-resistant FSGS (Table 3) with significant proteinuria and age-matched healthy controls. All samples were obtained with ethical approval (05/Q0508/6 Royal Free Hospital Research Ethics Committee (REC).

**Table 1:** Summary of treatments and clinical parameters of our index patient

Age	Presentation	Biopsy results	Action taken
15 mo	Nephrotic syndrome, acute kidney injury, negative immunology.	Mesangial proliferation, segmental glomerulosclerosis, nephrocalcinosis	Prednisolone (60 mg/m^2^/d) with no response. Cyclophosphamide (3 mg/kg/d) with partial remission
2 yrs	Relapse	N/A	Prednisolone and cyclophosphamide gave poor response. Addition of weekly vincristine (1.5 mg/m^2^) for 8 wks results in complete remission.
7 yrs	Relapse	N/A	Weekly vincristine for 8 wks results in complete remission
7 yrs 10 mo	Relapse	Varying degrees of segmental hyalinosis and sclerosis	Two-month course of weekly vincristine gave partial response
7 yrs to 11.5 yrs	Maintenance therapy	N/A	Vincristine every 2 wks, reducing to every 5–6 wks, then every 5–6 mo
15.5 yrs	Relapse	N/A	Complete response to 2-mo course of weekly vincristine
36 yrs	Relapse	N/A	Two-month course of weekly vincristine resulted in partial response. Relapse when decreasing the frequency. Further 4 mo of weekly, then alternate week vincristine followed by complete remission
40 yrs	Relapse	N/A	Two-mo course of weekly vincristine resulted in complete remission which was sustained
41 yrs	Remission	N/A	No further action

**Table 2:** Summary of renal parameters of each patient at the time of serum donation

Patient	Creatinine μmol/l	Albumin g/l	Urine protein-to-creatinine ratio mg/mmol	Treatment at time of sample collection
FSGS patient 1, sample 1: relapse no treatment (presentation)	110	25	1733	No treatment
FSGS patient 1, sample 2: during vincristine responding (treatment)	85	33	102	Weekly vincristine. Last dose of vincristine 1 wk before sample collection
FSGS patient 1, sample 3: in remission off treatment (remission)	81	44	19	Off all treatment. Last dose of vincristine 1 yr previously
FSGS patient 2	168	29	1255	Off all treatment
FSGS patient 3	105	23	1309	Prednisolone and cyclophosphamide
FSGS patient 4	102	34	676	Prednisolone and cyclosporin

FSGS, focal segmental glomerulosclerosis.

**Table 3:** Summary of clinical parameters of patients 2 to 4

Patient	Sex and age	Genetics	Biopsy results	Treatment at time of sample collection	Response
2	Female, 19 yrs	Homozygous *NPHS1* variant- c.1756A>G; p.(Arg586Gly)	Segmental sclerotic lesions	Off all immunosuppression at time of sample	No response to treatment- progression to ESRF
3	Female, 50 yrs	No genetics	Mesangial proliferation with early segmental lesions	Sample 15 d after 1.2 g i.v. cyclophosphamide and prednisolone 10 mg	Initial response to steroids, the steroid resistant with a minor or partial response to rituximab. CKD progression to ESRF
4	Male, 29 yrs	No genetics	No record	Prednisolone 3 mg and cyclosporin 100 mg twice a day	Progressive CKD with proteinuria. Died during follow-up; unknown cause of death

CKD, chronic kidney disease; ESRF, end-stage renal failure.

### Culture of Human Podocytes

A conditionally immortalized human podocyte cell line was cultured as described.^28^ For experiments, cells were exposed to RPMI-1640 medium, supplemented with either 10% fetal bovine serum (FBS) or patient serum with or without 5, 100, or 330 ng/ml vincristine for 24 hours.^15^ Vincristine doses were determined from pharmacokinetic data from patients treated with vincristine^29^ showing peak levels of 100 ng/ml reducing to 1 to 5 ng/ml after a few hours. Our highest dose was determined from studies showing the maximum bolus of vincristine administered to patients to be 2 mg^30^ with the average human having 5 l of blood. All experiments were independently repeated 4 times.

### Primary Glomerular Cell Isolation and GOAC

Human fetal kidney obtained from the Human Developmental Biology Resource (http://hdbr.org) at 23 postconceptional weeks with ethical approval from the National Health Service REC (23/LO/0312) was minced and digested at 37 °C. The digest was flowed through 100-μm sieves 3 times and glomeruli collected on a 30-μm sieve, plated and grown in a monolayer. Cells then underwent magnetic activated cell sorting to isolate podocytes (using biotin anti-podoplanin antibody, BioLegend, 337015, conjugated to antibiotin microbeads, Miltenyi Biotec, 130-090-485) and glomerular endothelial cells (gECs, using anti-CD31 microbeads, Miltenyi Biotec, 130-091-935) before expansion in VRADD (RPMI-1640, ThermoFisher, 218750910 supplemented with 10% FBS, 1% penicillin/streptomycin, 100 nM 1,25(OH)2D3, 1 μM all trans retinoic acid and 100 nM dexamethasone) and gECs (Cell Biologics, H1168) media, respectively. We generated the GOAC in OrganoPlate 3-lane 40-well plates (Mimetas) as described.^31^^,^^32^ On consecutive days, a collagen solution (5 mg/ml AMSbio Cultrex 3D Collagen-I rat tail, 1M HEPES, 7 g/l NaHCO_3_) matrix was first generated in the middle channel before podocyte and then gEC seeding (20,000 of each/microchip). The plate was left for 5 days and then exposed for 24 hours to glomerular gEC culture media containing either 1% FBS or presentation serum, with or without 5 ng/ml vincristine (*n* = 4) microchips/condition. Medium in the capillary channel was then replaced with serum free endothelial cell media containing 40 mg/ml FITC-albumin (Merck, A9771), and the noncellular channel medium replaced with serum-free RPMI. One hour later, the urinary space media was collected and FITC-albumin absorbance read at 485 nm.

### RNA Sequencing

RNA was extracted from podocytes exposed to presentation, treatment, or remission serums (*n* = 4 repeats/condition) using the RNEasy plus kit (Qiagen). Libraries were generated with the KAPA mRNA HyperPrep Kit (Roche, KK8580) with unique dual indexes and sequenced paired-end on the Illumina NextSeq 2000 platform with an average of 16 million reads per sample (ArrayExpress accession E-MTAB-14604). FASTq files were generated and pseudoaligned to the *Homo Sapiens* genome using Kallisto for Mac.^33^ Resulting count files, for each sample, were imported into Rstudio using the tximport package and the Ensembl gene IDs linked to each gene with the tx2gene() function.^34^ Genes with < 10 counts across all samples were removed. The DESeq2 program^35^ was used to determine differentially expressed genes with a statistical significance of *P* < 0.05 using the Wald test corrected for multiple comparisons between podocytes exposed to either treatment or remission serum versus cells stimulated with presentation serum. Resulting gene lists were ordered based on log2-fold change and separated into up and downregulated transcripts.

### Tubulin and F-Actin Immunolabelling

Podocytes were fixed and immunolabelled as described^15^ with either Acti-Stain 488 Phalloidin (Cytoskeleton Inc., 1:140) or anti-human α-tubulin (Abcam, ab6161, 1:200) followed by goat anti-mouse secondary antibody conjugated with 594nm fluorophore (Invitrogen, A11005, 1:200).

### IgG Removal and Western Blot

Presentation serum was passed through NAb Protein G Spin columns 3 times (ThermoFisher). Western blotting was undertaken as described^36^; samples were probed with mouse anti-human IgG antibody (1:1000, Novus Biologicals, NBP1-72758) followed by rabbit anti-mouse secondary antibodies (1:1000, Dako, P0260). Bands were detected using chemiluminescence.

### Image Analysis

F-actin^15^ and tubulin were analyzed blindly in 50 cells per condition for each independent experiment. The proportion of cells containing cortical actin stress fibers accumulating around the circumference of the cells was determined, whereas tubulin organization was classified into either being centrally nuclear concentrated or dispersed.

### Statistical Analysis

Statistical analyses were performed using GraphPad Prism 10.0 for Mac (GraphPad Software, Boston, MA). Data are presented as the mean ± SD and were assessed for normality using Shapiro-Wilk test. Statistical differences were analyzed by 1-way analysis of variance with Tukey’s multiple comparison tests for parametric data and Kruskal-Wallis test with Dunn’s multiple comparison test for nonnormally distributed data. Statistical significance was considered when *P* ≤ 0.05.

## Results

### Clinical History of our Index Patient With FSGS

Our index patient (Table 1, patient 1) was successfully treated with vincristine at Great Ormond Street Hospital during childhood, as previously described.^25^ Genetic screening detected no known pathogenic podocyte mutations. After 20 years of being in sustained remission, the patient relapsed at the age of 36 years, presenting with significant proteinuria (urine protein-to-creatinine ratio: 1733 mg/mmol), hypoalbuminemia (initially 25 g/l, decreasing to a nadir of 17 g/l), and acute kidney injury with serum creatinine peaking at 346 μmol/l. Weekly vincristine treatment was initiated, which resulted in a partial remission 4 months later (serum creatinine: 71 μmol/l, albumin: 36 g/l, urine protein-to-creatinine ratio: 173 mg/mmol). However, following a 2-week pause in vincristine therapy, there was a prompt relapse, resulting in reinitiation of weekly therapy and a slow wean over the subsequent 5 months. At this point, the patient had a creatinine of 87 μmol/l, albumin of 45 g/l and a urine protein-to-creatinine ratio of 55 mg/mmol. A complete remission was maintained until the age of 40 years when the patient relapsed once more (urine protein-to-creatinine ratio: 1293 mg/mmol, creatinine: 77 μmol/l, albumin: 27 g/l). Following a 2-month course of weekly vincristine, there was a complete response with a urine protein-to-creatinine ratio of 26 mg/mmol, albumin of 38 g/l and creatinine 78 μmol/l. At the latest consultation, just < 2 years later, the patient had normal renal parameters with a creatinine of 81 μmol/l, albumin of 44 g/l and a urine protein-to-creatinine < 20 mg/mmol.

### Vincristine Treatment Produces a Milieu Which Alters Podocyte Microtubule Genes

**Figure 1:**
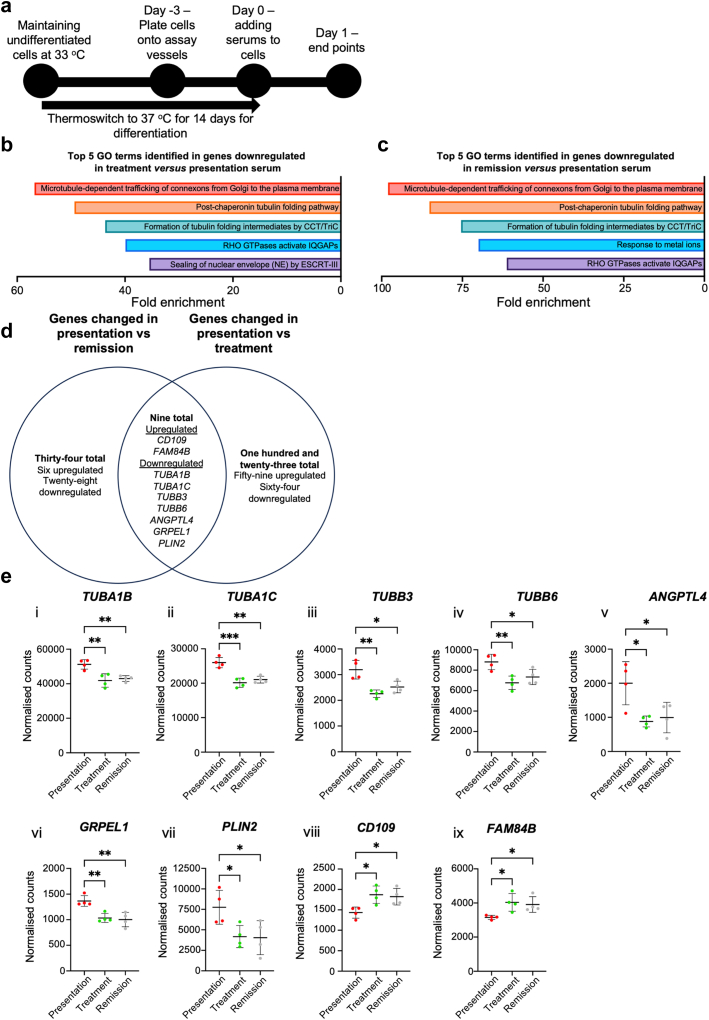
Vincristine alters the podocyte transcriptome in FSGS. (a) Schematic diagram demonstrating the experimental set up for all experiments. Top 5 gene ontology results derived from PANTHER Reactome Pathways with the input of downregulated podocyte genes compared with cells exposed to presentation serum in (b) cells exposed to serum taken during vincristine treatment, and in (c) cells exposed to serum taken when the patient was in remission. (d) Venn diagram summarizing differentially expressed genes analyses. (e) Normalized cell counts from all 3 treatment groups to show differential expression of (i) tubulin alpha 1b *(TUBA1B)*, (ii) tubulin alpha 1c *(TUBA1C)*, (iii) tubulin beta 3 class III *(TUBB3)*, (iv) tubulin beta 6 class VI *(TUBB6),* (v) angiopoietin-like 4 (*ANGPTL4*), (vi) GrpE like 1, mitochondrial *(GRPEL1)*, (vii) perilipin 2 (*PLIN2)*, (viii) cluster of differentiation 109 (*CD109)*, and (ix) family with sequence similarity 84, member B *(FAM84B)*. Data are shown as the mean ± SD of 4 independent experiments. For PANTHER Reactome Pathway analyses, the false discovery rate test was used to identify significantly enriched Reactome Pathways. For individual gene plots, 1- way analysis of variance with Tukey’s *post hoc* test was used. ∗*P* < 0.05, ∗∗*P* < 0.01, ∗∗∗*P* < 0.001. FSGS, focal segmental glomerulosclerosis; GO, gene ontology.

To understand how vincristine treatment might improve FSGS in our index patient, we examined the transcriptional profile of human immortalized podocytes^28^ exposed for 24 hours to sera (sample details in Table 2, patient 1) obtained when they (i) presented with disease, (ii) were undertaking vincristine treatment, and (iii) in remission (Figure 1a). For each condition, 17,633 genes were detected by our analysis. Principal Component Analysis revealed no distinct clustering between conditions (Supplementary Figure S1). We then compared the transcriptome between podocytes exposed to either presentation or vincristine treatment serum, finding 132 differentially expressed genes that were significantly (*P* < 0.05) altered (61 upregulated, 71 downregulated in the treatment group, Supplementary Table S1). In contrast, only 43 differentially expressed genes were detected in podocytes exposed to remission versus presentation serum (8 upregulated, 35 downregulated in the remission group, Supplementary Table S2).

We hypothesized that common biological processes might be identified in both of our comparative analysis and tested this using a gene ontology approach. From the transcripts significantly upregulated when podocytes were exposed to either vincristine treatment or remission serum compared with presentation serum, no pathways were identified by gene ontology analyses. In contrast, analysis of the downregulated podocyte transcripts, revealed that 4 of the top 5 biological pathways identified by gene ontology analysis were identical in the cells exposed to either vincristine treatment or remission serum (Figure 1b and c). Three of these pathways involved microtubules or their major constituent tubulin,^37^ with the fourth implicating another cytoskeletal protein,^38^ Ras homolog family member A.

We delved deeper into our gene lists and found 9 common transcripts altered when podocytes were exposed to either serum obtained during vincristine treatment or remission, compared with presentation serum (Figure 1d). In accord with our gene ontology analysis, 4 of these were tubulin isoforms (*TUBA1B, TUBA1C, TUBB3*, and *TUBB6*), whose levels were significantly reduced in podocytes exposed to either serum obtained during vincristine treatment or remission compared with presentation serum (Figure 1e, i–iv). In addition, significantly downregulated were transcript levels of angiopoietin-like 4 (Fig.1e, v), a secreted molecule whose overexpression in rat podocytes causes proteinuria^39^ alongside GrpE-like 1, mitochondrial (*GRPEL1*), a stress modulator in mammalian cells^40^ and the lipid droplet-binding protein,^41^ perilipin-2 (*PLIN2*) (Figure 1e, vi and vii). In contrast, elevated transcript levels of genes encoding cluster of differentiation 109 (*CD109,* Figure 1e, viii) (CD109) and the oncoprotein,^42^ family with sequence similarity 84, member 8 (*FAM84B,* Figure 1e, ix) were detected in podocytes exposed to either serum obtained during vincristine treatment or remission.

### Vincristine Treatment Alters Tubulin Distribution in Human Podocytes

**Figure 2:**
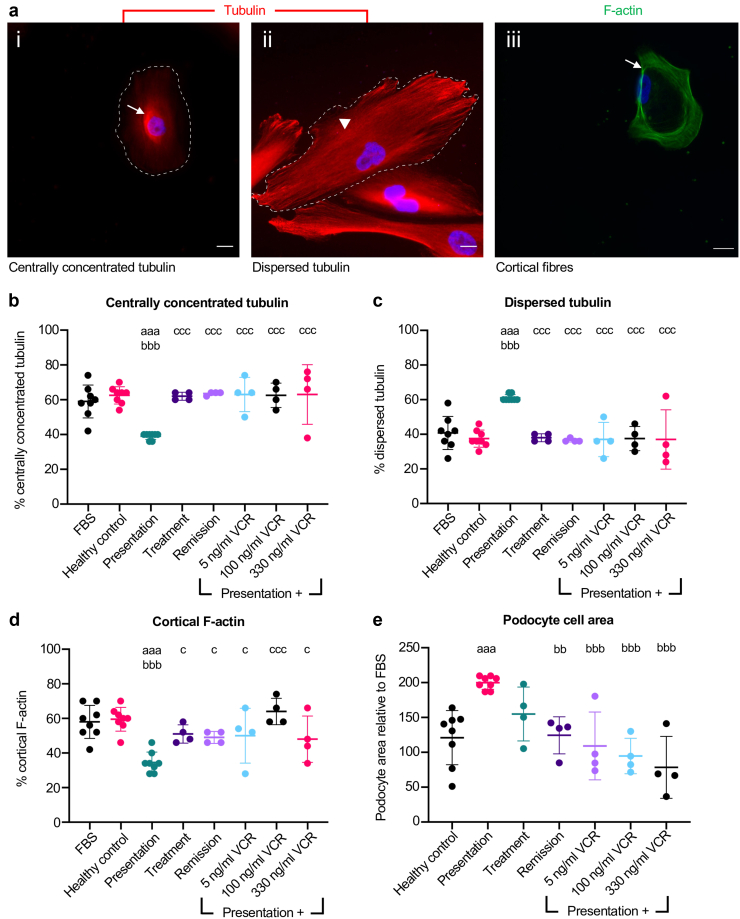
Vincristine protects the podocyte cytoskeleton and cell area in FSGS. (a) Representative images of podocyte (i) centrally concentrated tubulin (arrow), (ii) dispersed tubulin (arrowhead), (iii) cortical actin fibers (arrow). Prevalence of (b) centrally concentrated tubulin, (c) dispersed tubulin, (d) cortical actin fibers, and quantification of (e) podocyte cell area relative to FBS from biological repeat after exposure to either FBS, healthy control, presentation, treatment or remission serum as well as presentation serum with 5, 100, and 330 ng/ml of vincristine. One-way analysis of variance with Dunnett’s multiple comparison tests were performed. For (b), aaa = *P* < 0.001 versus healthy control, bb = *P* < 0.01 versus presentation, bbb = *P* < 0.001 versus presentation. For (c–e) aaa = *P* < 0.001 versus FBS, bbb = *P* < 0.001 versus healthy control c = *P* < 0.05 versus presentation, ccc = *P* < 0.001 versus presentation. Each data point shows the mean of 50 cells. Data are shown as the mean ± SD of 4 to 8 independent experiments. FBS, fetal bovine serum; FSGS, focal segmental glomerulosclerosis; VCR, vincristine.

Because both biological pathways involving microtubules and the levels of tubulin genes were altered in podocytes following exposure to serum obtained either during vincristine treatment or remission, we examined tubulin distribution. We quantified the amount of centrally nuclear-concentrated (Figure 2a, i; Figure 2b) and dispersed tubulin (Figure 2a, ii; Figure 2c) in 50 cells from 4 independent experiments. Podocytes exposed to FBS had a centrally concentrated and dispersed tubulin prevalence of 59.0% ± 9.4% and 41.0% ± 9.4%, respectively, with similar values obtained in cells treated with healthy control serum. Exposure of podocytes to presentation serum from our index patient 1 caused a shift to 39.0% ± 1.9% of centrally concentrated tubulin and 61.0% ± 1.9% dispersed tubulin (*P* < 0.001 vs. FBS and healthy control for both parameters). The prevalence of centrally concentrated tubulin was increased when podocytes were exposed to serum collected during either vincristine treatment (62.0% ± 2.3%) or remission (63.5% ± 1.0%) compared with the presentation serum (*P* < 0.001 for both comparisons). This corresponded with a reduction in dispersed tubulin (38% ± 2.3% and 36.5% ± 1.0% for vincristine treatment and remission serum, respectively) compared with the presentation serum (*P* < 0.001 for both comparisons). We also tested whether addition of vincristine to presentation serum altered tubulin distribution. Using 3 different doses, vincristine addition prevented the shift of centrally concentrated tubulin (5 ng/ml, 63.0% ± 9.9%; 100 ng/ml, 62.5% ± 7.0%; 330 ng/ml, 63.0% ± 17.2%) to dispersed tubulin (5 ng/ml, 37.0% ± 9.9%; 100 ng/ml, 37.5% ± 7.0%; 330 ng/ml, 37% ± 17.2%) seen in cells exposed to presentation serum (*P* < 0.001 for both parameters for all vincristine doses).

### Vincristine Treatment Results in Podocyte F-Actin Reorganization

Because microtubules and F-actin interact in response to changes in intracellular conditions,^43^ we next quantified the prevalence of cortical F-actin (Figure 2a, iii). From the results, 58.0% ± 9.6% and 59.5% ± 6.9 of podocytes exposed to FBS or healthy control serum, respectively, displayed a cortical F-actin pattern. Exposure to presentation serum from patient 1 significantly reduced the prevalence of cortical F-actin to 34.5% ± 6.0% (*P* < 0.001 compared with FBS and healthy control stimulated cells). Podocytes exposed to serum obtained either during vincristine treatment (51.0% ± 5.3%) or remission (49.0% ± 3.5%) caused a significant increase (*P* < 0.05 in both cases) in cortical actin stress fibers prevalence compared with cells exposed to presentation serum. This finding was replicated when either 5 ng/ml (50.0% ± 15.9%, *P* < 0.05 vs. presentation), 100 ng/ml (64.0% ± 7.6%, *P* < 0.001 vs. presentation), or 330 ng/ml (48.0% ± 13.4%, *P* < 0.05 vs. presentation) of vincristine was added to the presentation serum (Figure 2d).

We hypothesized that these cytoskeletal changes might result in alterations in podocyte size. Podocytes exposed to healthy control serum had a cell area of 121.0% ± 38.9% relative to FBS-stimulated cells. This significantly increased (*P* < 0.05) to 199.8% ± 9.5% when podocytes were exposed to the serum collected during presentation corresponding with the increase in the proportion of cells with dispersed tubulin and the reduction in the number of cells with a cortical F-actin pattern. There was a tendency for this increase in cell size to be ameliorated when podocytes were exposed to serum obtained during vincristine treatment, with a value of 155.0% ± 38.6% relative to FBS; however, this was not significantly different from cells stimulated with presentation serum. Podocytes exposed to serum obtained during remission had a cell area of 124.4% ± 26.6% relative to FBS, which was significantly lower than presentation (*P* < 0.01) serum stimulated cells. Furthermore, addition of either 5 ng/ml (109.2% ± 48.62%), 100 ng/ml (94.7% ± 25.4%), or 330 ng/ml (78.4% ± 44.0%) vincristine significantly reduced podocyte area compared with presentation serum alone (*P* < 0.001 for all doses) (Figure 2e).

Given that vincristine addition to presentation serum altered both tubulin distribution and F-actin organization, we tested whether vincristine exposure had any effect on healthy podocytes. However, we found that neither tubulin distribution (Supplementary Figure S2A and B), cortical F-actin (Supplementary Figure S2C), nor podocyte area (Supplementary Figure S2D) were changed in healthy podocytes following vincristine addition.

### F-Actin Reorganization in our Index Patient is not Because of the Presence of Circulating Autoantibodies

**Figure 3:**
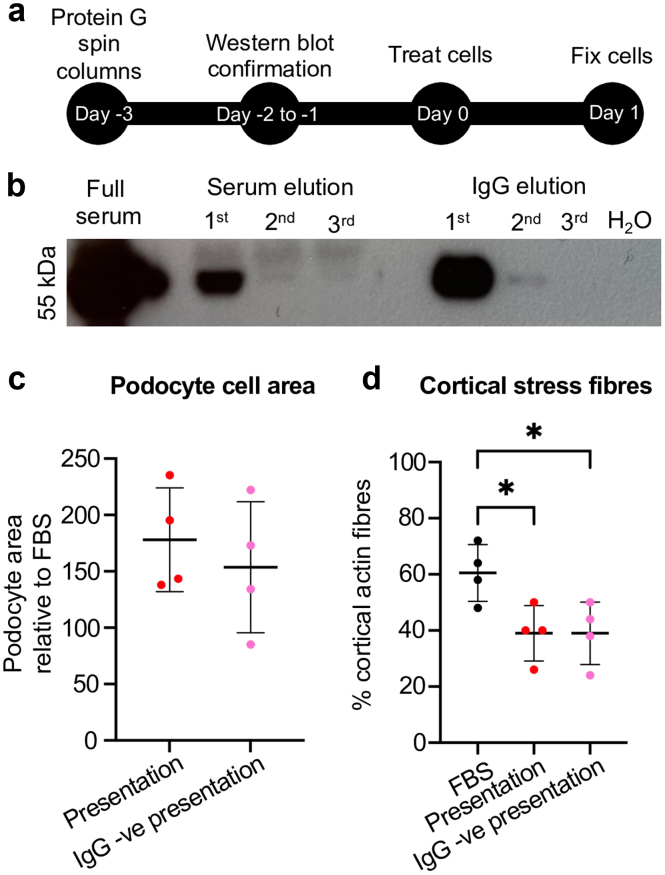
Podocyte F-actin reorganization following presentation serum exposure was not due to IgG in patient serum. (a) Experimental design. Serum was depleted of IgG before treatment of human immortalized podocytes with either presentation serum or IgG-depleted presentation serum. (b) Representative western blot of full serum, IgG depleted serum elutions and IgG elutions after 3 repetitions of protein G spin column IgG depletion. Quantification of (c) podocyte cell area (unpaired *t* test). Quantification of percentage of cells displaying (d) cortical F-actin stress fibers (1-way analysis of variance with Tukey *post hoc* test). Each data point shows the mean of 50 cells. Data are shown as the mean ± SD of 4 independent experiments. ∗*P* < 0.05. FBS, fetal bovine serum.

We hypothesized that the podocyte cytoskeletal reorganization following exposure to the presentation serum in our index case could be due to circulating autoantibodies directed against slit diaphragm proteins.^6^^,^^7^ To test this, we removed IgG from the presentation serum and assessed cortical F-actin podocyte distribution (Figure 3a). Western blotting showed that 3 cycles using protein G spin columns was sufficient to remove IgG from the presentation serum (Figure 3b).

After 24 hours of exposure, there was no significant difference in podocyte cell area (Figure 3c) when the cells were treated with either IgG-depleted or undepleted presentation serum (*n = 4* repeats/condition). Cells exposed to FBS showed a prevalence of 60.5% ± 10.1% cortical actin stress fibers (Figure 3d), which was significantly reduced to 39.0% ± 9.9% when podocytes were treated with undepleted presentation serum (*P* < 0.05). Similarly, podocytes exposed to IgG-negative presentation serum had a prevalence of 39.0% ± 11.1% cortical actin, which is significantly altered compared with FBS treated cells (*P* < 0.05), but not different compared with the undepleted presentation serum.

### Vincristine Addition to Serum From Patients With FSGS Prevents Albumin Permeability and Cytoskeletal Changes *in Vitro*

**Figure 4:**
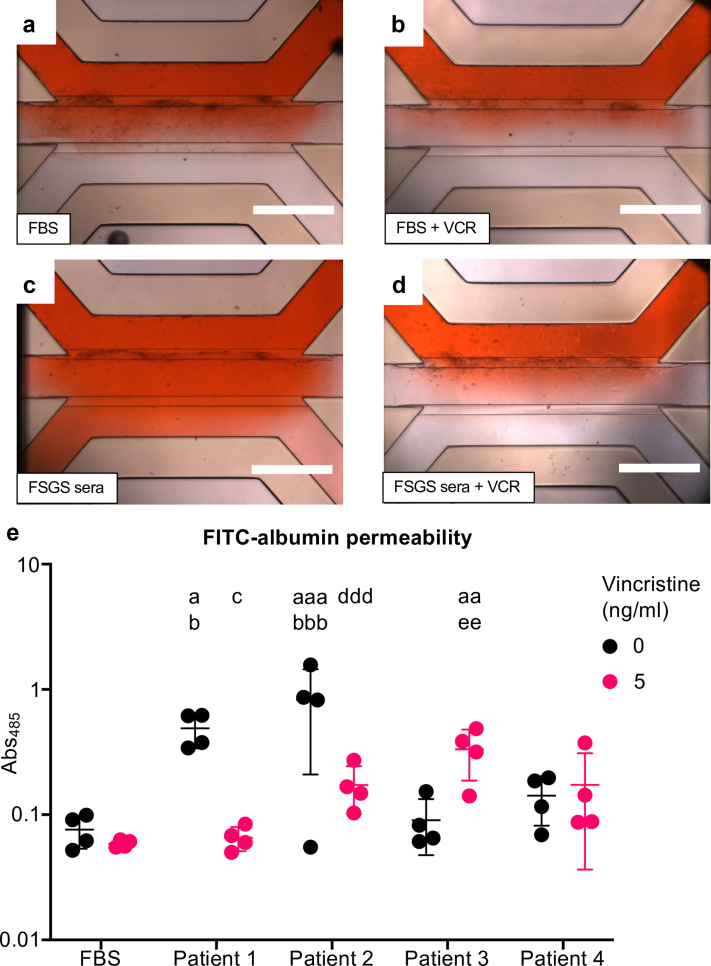
Vincristine addition to presentation serum from patients with FSGS reduced albumin permeability. Representative images of GOAC seeded with primary human podocytes and gECs from 23-week postconceptional kidneys exposed to (a) FBS, (b) vincristine, 1% presentation serum from 4 different patients with FSGS (c) without and (d) with 5 ng/ml vincristine. (e) Quantification of absorbance (485 nm) of FITC-albumin flow through into urinary space channel. Each data point represents a GOAC with 4 chips used for each condition. Scale bar = 750 μm. Data are shown as mean ± SD. Kruskal-Wallis test with Dunn’s multiple comparisons *post hoc* test were used for statistical testing. a, *P* < 0.05 versus FBS; aaa, *P* < 0.001 versus FBS; b, *P* < 0.05 versus FBS + 5 ng/ml; bbb, *P* < 0.001 versus FBS + 5 ng/ml; c, *P* < 0.05 versus patient 1; ddd, *P* < 0.001 versus patient 2. FBS, fetal bovine serum; FSGS, focal segmental glomerulosclerosis; gECs, glomerular endothelial cells; GOAC, glomerulus-on-a-chip; VCR, vincristine.

To determine if vincristine directly alters glomerular permeability, we used a GOAC^31^ model and performed FITC-albumin flow-through experiments following addition of either FBS (Figure 4a), 5 ng/ml vincristine (the lowest dose used in our earlier experiments, Figure 4b), 1% presentation serum from the index patient 1 with FSGS without (Figure 4c) or with 5 ng/ml vincristine (Figure 4d) for 24 hours (*n = 4* repeats/condition). Microchips exposed to FBS and vincristine showed no FITC-albumin flow-through into the urinary space channel, with Abs_485_ values of 0.08 ± 0.02 and 0.06 ± 0.004, respectively. Addition of presentation serum from patient 1 significantly increased Abs_485_ to 0.49 ± 0.15 (*P* < 0.05 vs. FBS and vincristine alone). Vincristine prevented this increase, with Abs_485_ of 0.07 ± 0.01 (*P* < 0.05 vs. presentation serum, Figure 4e).

We expanded our analysis to assess the effects of serum from 3 more patients with FSGS (Table 3) in the GOAC system. When added to the GOAC, the serum from patient 2, who had a genetic diagnosis of a homozygous nephrin mutation but was off all treatments when the sample was collected, resulted in an Abs_485_ of 0.82 ± 0.62, which was significantly higher than FBS addition (*P* < 0.001). Adding 5 ng/ml vincristine to this serum significantly reduced (*P* < 0.001, Figure 4e) the Abs_485_ to 0.17 ± 0.07. The genetics of patients 3 and 4 are unknown; however, both individuals were on steroid treatment at the time of serum collection. Serum from neither patient caused any change in FITC-albumin Abs_485_ compared with FBS, with values of 0.09 ± 0.04 and 0.14 ± 0.06, respectively. Adding 5 ng/ml vincristine to serum from patient 3 increased Abs_485_ to 0.33 ± 0.14, which was significantly higher than both FBS and serum alone (*P* < 0.01 in both cases). There were no significant differences when vincristine was added to serum from patient 4 (Figure 4e).

**Figure 5:**
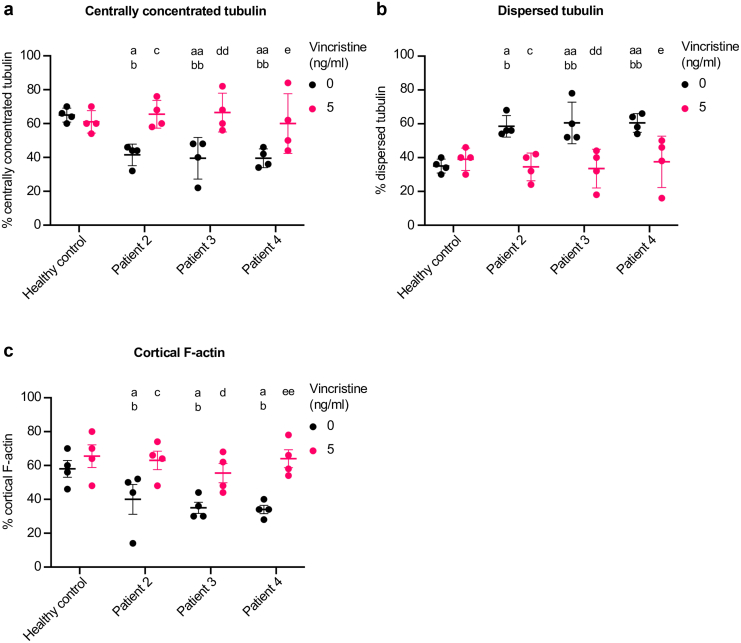
Podocyte tubulin disorganization was found following exposure to serum from multiple patients with FSGS. Quantification of (a) centrally concentrated tubulin, (b) dispersed tubulin, (c) cortical actin fibers from 3 other patients with FSGS with 5 ng/ml of vincristine. Each data point represents the mean of 50 cells and data are shown as the mean ± SD of 4 independent experiments. One way analysis of variance with Tukey’s *post hoc* test was used for all statistical testing. a, *P <* 0.05 versus healthy control; aa, *P* < 0.01 versus healthy control; b, *P <* 0.05 versus healthy control + 5ng/ml vincristine; bb, *P* < 0.01 versus healthy control + 5ng/ml vincristine; c, *P <* 0.05 versus patient 2 without vincristine; d, *P <* 0.05 versus patient 3 without vincristine; dd, *P <* 0.05 versus patient 3 without vincristine; e, *P <* 0.05 versus patient 4 without vincristine; ee, *P <* 0.05 versus patient 4 without vincristine.

Finally, we examined the effect of serum obtained from patients 2, 3, and 4 with and without vincristine addition on podocyte tubulin organization and F-actin distribution. Serum from patients 2, 3, and 4 reduced the prevalence of centrally concentrated tubulin (Figure 5a, *P* < 0.05 for patient 2, *P* < 0.01 for patients 3 and 4), but increased the number of podocytes displaying dispersed tubulin (Figure 5b, *P* < 0.05 for patient 2, *P* < 0.01 for patients 3 and 4) and lowered the prevalence of cells with cortical F-actin patterning in all patients (Figure 5c, *P* < 0.05 in all cases) compared with cells exposed to serum from healthy controls. The 5 ng/ml dose of vincristine protected podocytes from these changes, resulting in a significant increase in the prevalence of centrally concentrated tubulin (Figure 5a, *P* < 0.05 for patients 2 and 4, *P* < 0.01 for patient 3), a reduction in the number of cells displaying dispersed tubulin (Figure 5b, *P* < 0.05 for patients 2 and 4, *P* < 0.01 for patient 3), and an increase in the amount of cells with cortical F-actin patterning (Figure 5c, *P* < 0.05 for patients 2 and 3, *P* < 0.01 for patient 4).

## Discussion

Our study outlined the response to vincristine treatment for steroid-resistant FSGS in cultured human podocytes. Podocytes exposed to serum collected from our index patient with FSGS during either vincristine treatment or remission displayed reduced levels of genes associated with microtubule function alongside altered tubulin and F-actin patterning compared with cells stimulated with serum obtained during disease presentation. These structural changes were also prevented if vincristine was directly added to the presentation serum. We also showed that podocyte tubulin disorganization occurred when podocytes were exposed to serum obtained from 3 more patients with FSGS which could be prevented by vincristine. Furthermore, in some of our patients with FSGS, addition of serum to the GOAC led to increased albumin permeability, which could be prevented by vincristine addition. Collectively, our study is the first to link vincristine therapy with protective effects in human podocytes, in the context of FSGS.

We demonstrated that vincristine therapy can be successfully administered on multiple occasions during the relapses in FSGS to achieve a complete remission. Other studies (Supplementary Table S3) have reported successful vincristine treatment for pediatric FSGS.^25^^,^^26^^,^^44^^,^^45^ In the largest clinical trial to date, vincristine therapy for 8 weeks resulted in complete remission in 38.9%, and partial remission in 13.0% of 54 children with SRNS, without major side effects.^26^ In this cohort, 32 children had biopsy-proven FSGS, and 22 (68.75%) of these achieved remission after vincristine therapy.^26^ Our data suggest that vincristine can be considered for adult patients with FSGS unresponsive to conventional treatments.

Reduced levels of tubulin transcripts were found when podocytes were exposed to both vincristine treatment and remission sera compared with FSGS presentation serum. Similarly, retinal pigment epithelial cells exposed to combretastatin, another microtubule destabilizer, displayed reduced levels of tubulin transcripts, a finding attributed to phosphoinositide 3-kinase pathway inhibition.^46^ A similar mechanism may be involved in podocyte protection, a hypothesis supported by evidence showing that mutations in the F-actin–binding protein, anillin that cause FSGS were associated with hyperactivation of the phosphoinositide 3-kinase pathway.^47^ Increased cytosol levels of β-tubulins have also been linked with reduced β-tubulin mRNA levels as an autoregulatory response in Chinese hamster ovary cells.^48^ The shift in the patterning of microtubules from a dispersed to a centrally concentrated network by vincristine in podocytes could have increased the cytosolic concentration of tubulin, leading to a reduction in tubulin transcript levels.

Vincristine therapy reversed the changes in microtubules and F-actin seen when podocytes were exposed to presentation serum. Cytoskeletal alterations are associated with changes to podocyte polarity, cell shape, and foot process effacement^49^, ^50^, ^51^ likely resulting in the increase in FITC-albumin permeability seen with presentation serum from our index patient in the GOAC model, which was prevented by vincristine. Vincristine also attenuated albuminuria and foot process effacement in a rat model of FSGS.^52^ Here, the protective effect of vincristine was attributed to a3β1 integrin suppression^52^ and our RNA-sequencing data found that serum collected during vincristine treatment prevented a significant increase in podocyte integrin β1 that was present following exposure to FSGS presentation serum (Supplementary Table S1). We propose that the stabilization of both the microtubule and F-actin network by vincristine is likely to have prevented podocyte ultrastructural changes, including foot process effacement, thereby preventing albumin leakage in our index patient. We also found that serum from an individual with a homozygous nephrin mutation (patient 2) caused albumin leakage, indicating this patient not only had a podocyte gene variant, but also a circulating factor which may contribute to glomerular disease progression as has been postulated for some patients with nephrin mutations presenting with minimal-change disease.^53^ The podocyte changes induced by the serum could be prevented by vincristine. The serum from 2 other patients (patients 3 and 4) did not cause changes in albumin permeability despite causing podocyte alterations in tubulin and F-actin patterning. This may reflect the fact that these patients were on steroid treatment when serum samples were collected, which may dampen harmful circulating factors that contribute to changes in albumin permeability. Interestingly, vincristine addition to patient 3 increased albumin permeability, suggesting that the effect of vincristine treatment might be patient-dependent and not be beneficial in all FSGS cases.

There are several potential explanations for how vincristine might protect podocytes. Recent studies have indicated that circulating autoantibodies against the slit diaphragm protein nephrin cause some FSGS cases.^6^^,^^52^, ^53^, ^54^ Although, vincristine can have immunosuppressive effects and inhibit antibody formation,^54^^,^^55^ it is unlikely this is the mechanism of action in our index patient, because we found no differences in the cytoskeletal changes induced by presentation serum following IgG depletion. Alternatively, vincristine treatment might cause changes in the patient’s circulating milieu. Supporting this idea, we found that podocyte *ANGPTL4* was significantly reduced following exposure to serum obtained during and after vincristine treatment and increased levels of this factor are associated with proteinuria in patients with diabetic nephropathy,^56^ SRNS,^39^ and membranous nephropathy.^57^ Furthermore, transgenic overexpression of *Angptl4* in rat podocytes increased proteinuria and foot process effacement.^39^ Soluble urokinase plasminogen activator receptor (suPAR) is another proposed damaging circulating factor in FSGS,^58^ which binds to podocyte plasminogen activator receptor and modulates the activity of the cytoskeletal proteins Ras homolog family member A and Rac1 in a cellular human podocyte model of FSGS.^59^ Our transcriptional results show elevated *PLAU* (urokinase-type plasminogen activator) levels, the secreted natural ligand for uPAR, in podocytes exposed to presentation serum compared with the treatment serum; and this warrants further investigation. An alternative explanation is that vincristine directly affects podocytes, supported by our data showing that direct vincristine addition to FSGS presentation serum protected podocytes from the serum-induced changes in tubulin and F-actin and attenuated albumin leakage seen in the GOAC in 2 of the patients with FSGS examined. It is also possible that vincristine affects gECs, which may contribute to the changes seen in albumin leakage in our GOAC experiments.

In conclusion, we provide the first evidence that vincristine therapy protects against pathological changes induced by FSGS serum, associated with the preservation of tubulin and F-actin organization. Furthermore, we demonstrate the effectiveness of vincristine in adult steroid resistant FSGS, warranting further investigation to advance our understanding of this alternative FSGS therapeutic.

## Disclosure

All the authors declared no competing interests.

## Supplementary Material

Supplementary File (PDF) **Figure S1.** Whole transcriptome clustering after addition of sera. **Figure S2.** Vincristine has no effect on podocyte cell area, tubulin, or F-actin. **Table S1.** Genes significantly altered in podocytes exposed to treatment versus presentation serum. **Table S2.** Genes significantly altered in podocytes exposed to treatment versus remission serum. **Table S3.** Summary of studies administering vincristine in patients with glomerular diseases.
