# Supplementary material for: Vincristine Treatment Protects Against Podocyte Damage in Focal Segmental Glomerulosclerosis
Source: Kidney Int Rep. 2025 Aug 14;10(11):3998–4011. doi: 10.1016/j.ekir.2025.08.002 (PMC12640030; doi:10.1016/j.ekir.2025.08.002)
Supplement: Supplementary File (PDF) — Figure S1. Whole transcriptome clustering after addition of sera. Figure S2. Vincristine has no effect on podocyte cell area, tubulin, or F-actin. Table S1. Genes significantly altered in podocytes exposed to treatment versus presentation serum. Table S2. Genes significantly altered in podocytes exposed to treatment versus remission serum. Table S3. Summary of studies administering vincristine in patients with glomerular diseases. [file mmc1.pdf]

Supplementary Material

Vincristine treatment protects against podocyte damage in focal segmental glomerulosclerosis

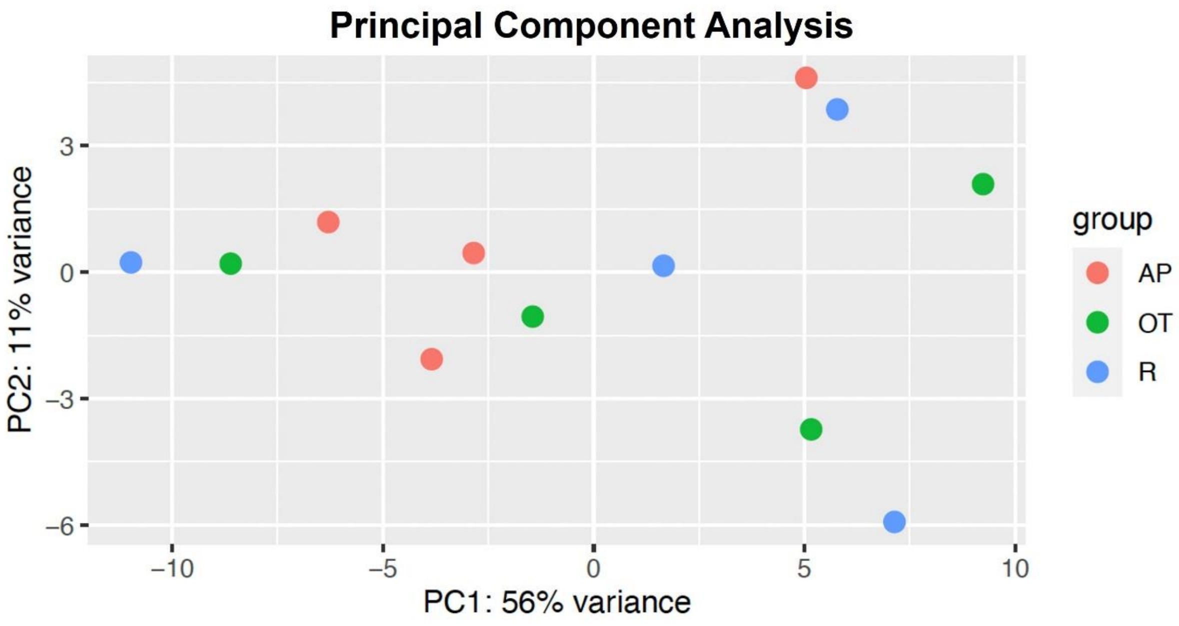

Supplementary Figure 1. Whole transcriptome clustering after addition of sera. Principal component analysis (PCA) of human immortalised podocytes treated with presentation (AP), vincristine treatment (OT) and remission (R) sera after bulk RNA sequencing. Each dot represents one biological repeat.

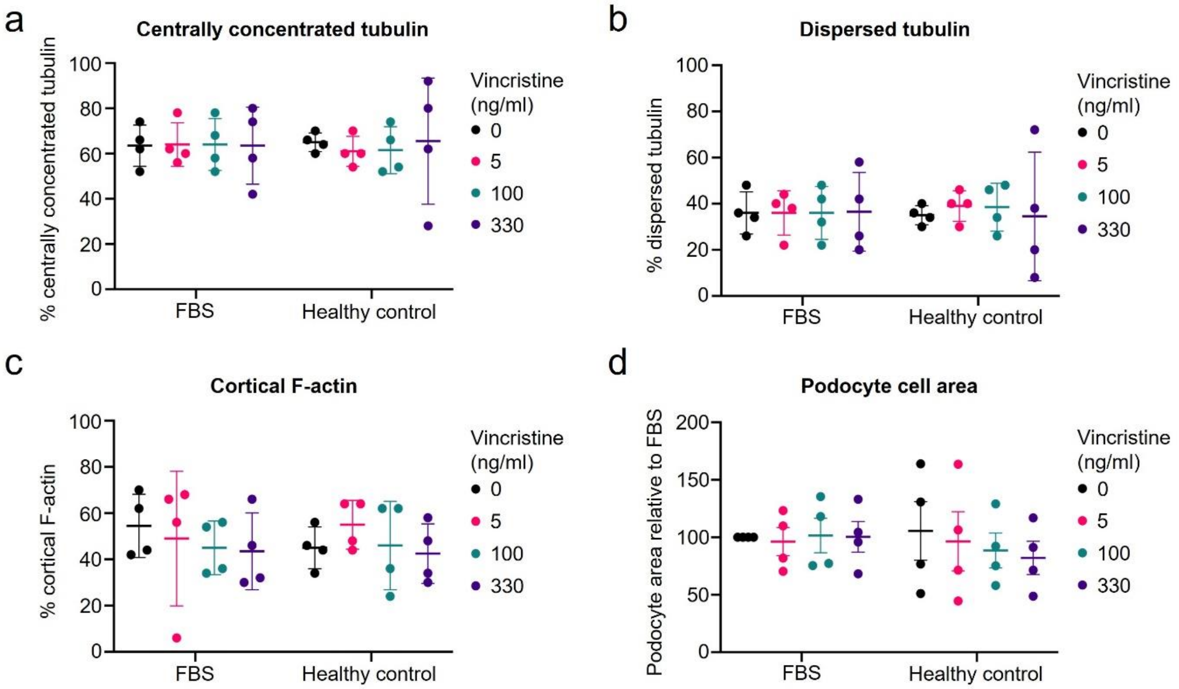

Supplementary Figure 2. Vincristine has no effect on podocyte cell area, tubulin, or F-actin. Prevalence of (a) centrally concentrated tubulin, (b) dispersed tubulin and (c) cortical F-actin. (d) Quantification of podocyte cell area relative to FBS from biological repeat. Two-way ANOVA with Tukey's multiple comparison tests were performed. Each data point shows the mean of 50 cells. Data are shown as the mean ±SD of 4 independent experiments.

Table S1

Genes significantly downregulated in podocytes exposed to treatment *versus* presentation serum

Downregulated in treatment serum	baseMean	log2FoldChange	lfcSE	stat	pvalue	padj	FoldChange
ID1	148.1114099	-1.270618803	0.211438697	-6.009395723	1.86216090328689E-09	1.66330872111447E-06	0.414481955
ANGPTL4	1290.74808018736	-1.104956604	0.265784396	-4.157341888	3.21971964539643E-05	0.007189749	0.464916456
PLIN2	5240.98378325677	-0.825520763	0.232429384	-3.55170568	0.000382743	0.040560998	0.56427848
VARS	513.8921918	-0.789813517	0.214776238	-3.677378482	0.000235643	0.028075251	0.578418853
MT-CO3	157498.677022884	-0.774243979	0.090226832	-8.581083536	9.39835962146915E-18	1.67894981523531E-14	0.584694944
G0S2	317.7341621	-0.739060439	0.204450345	-3.614865202	0.000300504	0.033324905	0.599129411
MT-CYB	66838.089982827	-0.687829825	0.190304358	-3.614367175	0.000301082	0.033324905	0.620786967
PNMA2	221.6836343	-0.665087608	0.169964878	-3.913088488	9.11231239240049E-05	0.014243683	0.630650407
HILPDA	470.8787153	-0.650568996	0.178443423	-3.6457998	0.000266561	0.031152812	0.637029021
PDK4	267.0623854	-0.622300956	0.165467524	-3.760864622	0.000169327	0.022525903	0.649633999
MT-CO2	86642.5354649145	-0.616255725	0.106342245	-5.795022731	6.83119280573736E-09	5.02494506092621E-06	0.652361829
HMOX1	398.4995836	-0.600635449	0.149894999	-4.007041284	6.14841193544925E-05	0.010532314	0.659463424
PECR	168.5222087	-0.563892663	0.15705499	-3.590415442	0.000330151	0.036215283	0.676474444
NRCAM	418.76704	-0.532004194	0.132134471	-4.026233218	5.6677476673606E-05	0.00998242	0.691593304
ARRDC4	1548.20251841468	-0.530261086	0.107636728	-4.926395434	8.37604031904746E-07	0.000376282	0.692429413
TUBB3	2658.10370490507	-0.492501076	0.082691013	-5.955920205	2.58612497312418E-09	2.02121829930737E-06	0.710791791
RPL22L1	453.0008529	-0.490490244	0.108816541	-4.507497098	6.55968276374384E-06	0.001953067	0.711783185
BOLA2B	322.7222457	-0.488360437	0.133585505	-3.655789117	0.000256392	0.030247007	0.712834745
EDN1	2044.41729712518	-0.459463651	0.074333987	-6.181071002	6.36681711137402E-10	6.63475399814434E-07	0.72725658
TRIT1	359.82253	-0.450439636	0.119118338	-3.781446604	0.00015592	0.021426104	0.731819805
RRAGA	3754.01284683459	-0.44964245	0.095880651	-4.689605731	2.7373196566714E-06	0.00092514	0.732224296
IGFBP3	6290.44644756295	-0.443807226	0.084634301	-5.243822198	1.57283774923202E-07	0.000103518	0.735191899
DOCK7	1287.26053545458	-0.443609844	0.127766816	-3.472027076	0.000516544	0.04856681	0.73529249
FARP2	752.8677516	-0.435866894	0.118529824	-3.677276129	0.000235738	0.028075251	0.739249417
AC010970.1	41192.1366594802	-0.429469344	0.111135169	-3.864387386	0.000111368	0.016983674	0.742534856
KRT34	533.8369988	-0.426315767	0.113348758	-3.761097832	0.000169169	0.022525903	0.744159733
TSTA3	996.7154113	-0.409462858	0.10394782	-3.93911925	8.17812763600635E-05	0.013281492	0.752903641
TRA2A	993.9784501	-0.401424548	0.113870567	-3.525270484	0.000423051	0.043362686	0.757110327
GRPEL1	1133.71345922263	-0.400851158	0.087056271	-4.604506393	4.13445316575924E-06	0.001292533	0.757411296
MT-ND3	22783.2752211245	-0.400515647	0.08642004	-4.634522793	3.57761815433656E-06	0.001147131	0.757587459
ASL	845.0408557721	-0.387414213	0.094881947	-4.083118278	4.44353920707E-05	0.009109255	0.764498612
DESI1	780.0350469	-0.381591707	0.089693114	-4.254414758	2.09596484744257E-05	0.005139224	0.767590251
TUBB6	7637.61290409054	-0.379731695	0.057015903	-6.660101432	2.73638667318141E-11	3.42185153481336E-08	0.768580514
ORMDL2	1711.52881174309	-0.371430138	0.085535078	-4.342430562	1.4091502997224E-05	0.003765697	0.773015829
TUBA1C	22399.2111944563	-0.367027821	0.049469786	-7.419232009	1.1780139948925E-13	1.84138312576635E-10	0.775378254
KLF10	1459.7282883008	-0.356131547	0.101648029	-3.503575548	0.000459056	0.044959038	0.781256641
SRSF2	1730.6885381432	-0.351257909	0.066403706	-5.289733526	1.22494692668465E-07	8.50997851010641E-05	0.783900304
CD2BP2	1304.4086863067	-0.341729909	0.098382446	-3.473484591	0.000513747	0.04856681	0.789094555
SPTLC2	2460.29166596655	-0.340947391	0.088588483	-3.848664989	0.000118763	0.017680179	0.789522677
TUBA4A	2944.933012397	-0.336030595	0.083206332	-4.03852192	5.37890677565536E-05	0.00998242	0.79221801
TXNIP	5292.12059413769	-0.335165631	0.0832235	-4.027295539	5.64220946727349E-05	0.00998242	0.792693125
HSPA8	45373.1734351525	-0.329546885	0.06580461	-5.007960444	5.50098360146309E-07	0.000286624	0.795786381
TUBB4B	5652.28903577887	-0.317052288	0.078649463	-4.031207264	5.54910810239435E-05	0.00998242	0.802708295
AMD1.00	2496.86189607162	-0.313729351	0.075732134	-4.142618657	3.43362617498049E-05	0.007532894	0.804559292
NQO1	3141.9061659083	-0.309020981	0.069845647	-4.424341293	9.67369876980712E-06	0.002749309	0.807189336
TUBA1A	35031.623241181	-0.308964151	0.051562626	-5.99201738	2.07253769663978E-09	1.7278055930987E-06	0.807221132
ACAA1	1214.3460913566	-0.306373404	0.079571214	-3.850304504	0.000117971	0.017680179	0.808672017
C8orf33	1078.16548766722	-0.305964926	0.080143014	-3.817736688	0.000134682	0.019043516	0.808901014
HBEGF	889.30849892974	-0.301114922	0.080931795	-3.720601083	0.000198749	0.024853584	0.811624926
DYNLL1	7548.19215222369	-0.29988619	0.058282287	-5.14540875	2.6693893370177E-07	0.000165568	0.812316475
TUBA1B	45364.4250025756	-0.292806288	0.058805328	-4.979247614	6.38319334694107E-07	0.000319287	0.816312646
PSMA5	2973.4089491838	-0.287562098	0.071086295	-4.045253683	5.22664949616612E-05	0.00998242	0.819285338
KRT18	6041.18907763786	-0.27830341	0.057752984	-4.818857719	1.4438246868853E-06	0.00056422	0.824560119
EIF5A	12760.6648511981	-0.269374445	0.056344192	-4.780873349	1.74535298188668E-06	0.000641931	0.829679218
ACADVL	2136.2513955854	-0.269082384	0.06276901	-4.2868668	1.81210874891281E-05	0.004532084	0.829847197
TPM3	13153.655376535	-0.266166851	0.070963485	-3.750757886	0.000176301	0.022728273	0.831525925
EDF1	3021.09865673778	-0.252574908	0.059761079	-4.226411428	2.37447672534183E-05	0.005498672	0.83939693
MOK	3391.24936350128	-0.251212802	0.071423376	-3.517235061	0.000436067	0.044333522	0.840189812
CTPS1	2811.0190126848	-0.24734887	0.06295802	-3.928790469	8.53741854440124E-05	0.013513977	0.842443087
TUBB2A	5351.3600586741	-0.244903276	0.06519782	-3.756310832	0.000172437	0.022619881	0.843872371
COTL1	10230.3071448139	-0.238122589	0.046347613	-5.137753075	2.78042888828808E-07	0.000165568	0.847847918
NNMT	15434.2950170925	-0.23250098	0.060841924	-3.821394248	0.000132699	0.019043516	0.851158089
TAGLN2	13646.2848366228	-0.225832834	0.055551191	-4.065310399	4.79685773414831E-05	0.009521382	0.855101253
S100A10	11338.9139465101	-0.224168706	0.064370487	-3.48247647	0.000496799	0.047788231	0.856088169
EIF4A1	19839.186888958	-0.214337427	0.059987076	-3.573060088	0.000352834	0.038366836	0.861941925
CAP1	28038.2860361565	-0.205062092	0.046454895	-4.414219261	1.01375174406022E-05	0.002817103	0.86750135
ACTG1	87357.9129197786	-0.200140007	0.055374177	-3.614320224	0.000301137	0.033324905	0.870466084
PPP2R1A	5026.26829185385	-0.194251941	0.055304155	-3.512429408	0.00044403	0.044420756	0.874025974
ANXA1	11879.2139347617	-0.19118687	0.051224569	-3.732327518	0.000189719	0.023963951	0.875884856
TPM1	41086.058594606	-0.184683001	0.052173392	-3.539792852	0.000400441	0.041384441	0.87984238
CSRP1	9853.9028128187	-0.184676027	0.052067775	-3.546839202	0.000389882	0.040924421	0.879846633

Genes significantly upregulated in podocytes exposed to treatment *versus* presentation serum

Upregulated in treatment serum	baseMean	log2FoldChange	lfcSE	stat	pvalue	padj	FoldChange
ITGB1	39477.2080026616	0.266657641	0.054140958	4.92524789	8.42534753874478E-07	0.000376282	1.20301751
SORT1	4012.05526506707	0.278963973	0.060660663	4.598762385	4.25008191878676E-06	0.001296275	1.213323261
IGFBP7	10396.3522952345	0.282700235	0.071864541	3.933793083	8.3615771851851E-05	0.013405323	1.216469573
PLAU	4007.36144963705	0.287247798	0.066343748	4.329689033	1.49320046899777E-05	0.003810709	1.220310093
SLAIN2	1250.52001537989	0.309091281	0.076448927	4.043108174	5.27472698614839E-05	0.00998242	1.238927084
CCNI	6018.46880506708	0.314339724	0.079754755	3.941328951	8.10314091152277E-05	0.013281492	1.243442439
MME	1630.63093884021	0.321363151	0.090408917	3.554551516	0.000378624	0.040467498	1.249510609
OSTM1	2156.50687180717	0.324382296	0.086812188	3.736598556	0.000186526	0.023801144	1.252128213
TIMELESS	756.7171592	0.333264101	0.091813055	3.629811715	0.000283628	0.032539161	1.25986059
ANKRD1	2537.82687530797	0.338216807	0.083192307	4.065481745	4.79333463053964E-05	0.009521382	1.264193066
C5orf24	2237.90279925598	0.346769374	0.098699839	3.51337324	0.000442455	0.044420756	1.271709698
FAM84B	3701.80549505765	0.349715001	0.08802065	3.973101797	7.09427118159698E-05	0.011828515	1.274308868
KLHL13	1578.91439512815	0.361748756	0.08353913	4.330291131	1.48912334596733E-05	0.003810709	1.284982539
GNPDA1	1996.72054637727	0.379022043	0.092144067	4.11336352	3.89935613243891E-05	0.008264652	1.300460016
CD109	1692.5768419741	0.383649531	0.078875051	4.86401628	1.15027446288706E-06	0.000466888	1.304637978
LCP1	591.0531584	0.388508239	0.102442162	3.792464277	0.00014916	0.020724913	1.309039145
OXTR	1343.81190356129	0.400890602	0.114444859	3.502914893	0.000460196	0.044959038	1.320322719
HERC5	589.1947465	0.403370329	0.115409394	3.495125601	0.000473839	0.04593297	1.322594062
ZNF302	1343.77970108202	0.442464708	0.115731637	3.823195797	0.000131733	0.019043516	1.358923938
VTI1A	658.4096277	0.447353225	0.116512477	3.839530619	0.00012327	0.018135156	1.36353641
NOMO2	1097.40102088765	0.447407783	0.108110847	4.138417136	3.49710130975377E-05	0.007539871	1.363587976
PDP1	1293.24303591908	0.449141568	0.107545066	4.176310322	2.96275572563811E-05	0.006736229	1.365227678
RASEF	263.4956257	0.461918298	0.127634083	3.61908267	0.000295649	0.033324905	1.377372041
ANKRD13C	638.3755538	0.522034477	0.143582986	3.635768352	0.000277153	0.03209076	1.435978827
FAM49A	1144.5479035886	0.523590659	0.102235829	5.121400836	3.03274211883515E-07	0.000167302	1.4375286
CBLB	219.2131751	0.56948423	0.154254725	3.691843017	0.000222635	0.027037486	1.483992942
TUT7	330.788607	0.593027952	0.157949024	3.754552818	0.000173651	0.022619881	1.508409302
TSC2	298.3686105655	0.603090906	0.170127733	3.54493001	0.000392717	0.040924421	1.518967398
PPARA	410.03085	0.623046643	0.131564901	4.735660025	2.18343342226708E-06	0.00078011	1.540124141
FAM129A	617.3348705	0.624624056	0.12202879	5.11866139	3.07711854478676E-07	0.000167302	1.541809002
TMEM86A	391.1512604	0.652293995	0.13972331	4.668469395	3.03451970310312E-06	0.000998597	1.571665277
FGF23	1326.00681256175	0.657203687	0.095658557	6.870307355	6.40637241158991E-12	8.90129855632576E-09	1.577022984
GPM6A	587.9757908	0.66206736	0.152498484	4.341468473	1.41533589554334E-05	0.003765697	1.582348481
LSAMP	151.8783588	0.674457411	0.189450351	3.560074756	0.000370749	0.039967407	1.595996415
PLD1	289.389606	0.692188354	0.144545466	4.788724077	1.67845074269975E-06	0.000636031	1.615732486
SNTB2	403.3771724	0.69390292	0.171111356	4.055271	5.0076211128381E-05	0.009784422	1.61765384
CXCL14	150.7892019	0.736720262	0.173881711	4.236904845	2.266221195665E-05	0.005449826	1.666383272
GUCY1A2	207.681344	0.745550926	0.195365949	3.816176405	0.000135536	0.019043516	1.676614401
TGFBR3	474.6825108	0.748969485	0.153626485	4.875262776	1.08663708944154E-06	0.000466888	1.680591957
DOCK9	543.694719	0.766564289	0.203499101	3.766917332	0.000165276	0.02246492	1.701213595
NEUROD2	308.9219709	0.785711711	0.176168179	4.460009276	8.1956105766461E-06	0.002383398	1.723942566
ZBTB20	431.46322083076	0.813298513	0.1923847	4.227459421	2.36344781752005E-05	0.005498672	1.757224493
AHR	301.1238218	0.824640232	0.206090787	4.001344467	6.29835891245915E-05	0.010643375	1.771093322
JMJD1C	174.59009486943	0.833762314	0.207435903	4.019373223	5.83531707114978E-05	0.010134811	1.782327326
MTRNR2L10	4134.92227811574	0.851711848	0.080569531	10.57114069	4.05531689013374E-26	1.69039125703741E-22	1.804640975
MTRNR2L11	11063.094291604	0.856080529	0.087063553	9.832823209	8.13068642792124E-23	1.69457056301925E-19	1.810113961
TERT	343.8602171	0.866288808	0.178146406	4.862791385	1.15741813670701E-06	0.000466888	1.822967458
NIPAL4	94.85359643	0.868461861	0.249813743	3.476437485	0.000508123	0.048504382	1.825715363
ZNF516	171.4344353	0.87143018	0.224213291	3.886612496	0.000101653	0.01569344	1.829475607
MTRNR2L1	29073.7787237528	0.891214434	0.086975169	10.24676867	1.22366085030283E-24	3.06037578660738E-21	1.85473675
MTRNR2L8	60772.3920208379	0.891264293	0.085812474	10.38618575	2.86585730148641E-25	8.9593863887719E-22	1.85480085
MTRNR2L12	238026.188054719	0.92113323	0.078104568	11.7935897	4.21197514066813E-32	5.267074913405E-28	1.893602124
MT1L	325.6871386	0.922990986	0.139033649	6.638615848	3.16642432972949E-11	3.59964874938794E-08	1.896042084
MTRNR2L4	6352.23027191471	0.931116062	0.08616998	10.80557363	3.23925971864281E-27	2.02534713908142E-23	1.906750479
AL355315.1	88.57503152	0.963348887	0.274741715	3.506380121	0.000454246	0.044959038	1.949830732
RASGRP3	98.95926687	1.013577991	0.273771133	3.70228219	0.000213669	0.026454733	2.018911948
MT1F	205.575827	1.212307174	0.196661375	6.164439622	7.07331790605358E-10	6.80398772424616E-07	2.317078904
STAT5A	96.79897265	1.257398724	0.265902333	4.728799143	2.25851675650673E-06	0.000784521	2.390643031
NCAM2	45.22909368	1.352777073	0.330995784	4.086991848	4.37002467564285E-05	0.00910786	2.554032836
GON4L	1229.88283068039	1.422169714	0.352640537	4.032916137	5.50889432698602E-05	0.00998242	2.679882438
RAB11FIP4	28.83706575	1.531093814	0.414731763	3.691768871	0.0002227	0.027037486	2.89004872
AC129492.1	35.89638523	1.737968207	0.349939215	4.966485983	6.81769785625353E-07	0.000327905	3.335650666

Table S2

Genes significantly downregulated in podocytes exposed to treatment *versus* remission serum

Downregulated in remission serum	baseMean	log2FoldChange	lfcSE	stat	pvalue	padj	FoldChange
**ANGPTL4**	1290.74808	-1.066624406	0.265735733	-4.013853885	5.97E-05	0.007963806	0.477434787
**PLIN2**	5240.983783	-0.985000229	0.232441839	-4.237620181	2.26E-05	0.004732637	0.505225643
**MT2A**	2781.321456	-0.889701667	0.133361042	-6.671376072	2.53E-11	7.43E-08	0.539725716
**AC010970.1**	41192.13666	-0.610840329	0.111138362	-5.49621498	3.88E-08	2.85E-05	0.65481518
**HMGA2**	1770.226739	-0.501570803	0.131552446	-3.812706032	0.000137454	0.016126052	0.706337304
**GRPEL1**	1133.713459	-0.452772391	0.086882449	-5.211321658	1.88E-07	0.000109988	0.730637451
**TXNRD1**	19819.5272	-0.434705626	0.105628222	-4.115430688	3.86E-05	0.006399761	0.7398447
**MOK**	3391.249364	-0.412669929	0.071505121	-5.771194081	7.87E-09	7.7E-06	0.751231817
**LOX**	2912.400423	-0.398577008	0.105132748	-3.79117844	0.000149934	0.016913729	0.75860616
**CACYBP**	1705.173041	-0.378341412	0.104498295	-3.620551039	0.000293976	0.02973215	0.76932153
**TUBB3**	2658.103705	-0.340286218	0.08246342	-4.126511086	3.68E-05	0.006399761	0.78988459
**RRAGA**	3754.012847	-0.327578641	0.095758647	-3.420877915	0.000624193	0.047333303	0.7968728
**PLP2**	5003.374192	-0.320337567	0.067625958	-4.736902503	2.17E-06	0.00074347	0.800882462
**TUBA1C**	22399.21119	-0.303440629	0.049440937	-6.137436806	8.39E-10	1.23E-06	0.810317594
**FKBP4**	2548.783507	-0.301270982	0.065519429	-4.598193027	4.26E-06	0.001136327	0.811537136
**CTPS1**	2811.019013	-0.297174121	0.062870638	-4.726755293	2.28E-06	0.00074347	0.813844955
**NQO1**	3141.906166	-0.286840943	0.069762137	-4.111699518	3.93E-05	0.006399761	0.819694974
**ODC1**	2491.31174	-0.285559241	0.084073483	-3.396543479	0.000682427	0.047333303	0.820423521
**UBASH3B**	1655.340622	-0.283223263	0.079228741	-3.574754046	0.000350557	0.032130766	0.821753008
**CYCS**	2928.885292	-0.268128405	0.07873668	-3.405381149	0.000660718	0.047333303	0.830396112
**GAPDH**	121720.5819	-0.267782151	0.078891408	-3.394313221	0.00068801	0.047333303	0.830595436
**TUBB6**	7637.612904	-0.26333973	0.056912244	-4.627119087	3.71E-06	0.001087519	0.83315699
**ITGB1BP1**	2493.2954	-0.251896399	0.074237099	-3.393133671	0.000690979	0.047333303	0.839791797
**TUBA1B**	45364.425	-0.2518559	0.058795768	-4.283571908	1.84E-05	0.004149442	0.839815372
**EIF5A**	12760.66485	-0.250862129	0.056318067	-4.454381013	8.41E-06	0.002056414	0.84039406
**TACC1**	2425.874442	-0.237145319	0.070044102	-3.385657206	0.00071008	0.047333303	0.848422438
**COTL1**	10230.30714	-0.231592491	0.046308761	-5.001051298	5.7E-07	0.000278726	0.851694247
**EIF2S1**	3558.547377	-0.221903944	0.064218746	-3.455438782	0.000549398	0.046039547	0.857433123
**KRT19**	11450.85762	-0.22184737	0.059447251	-3.73183564	0.00019009	0.019911877	0.857466747
**TXN**	5337.227402	-0.221776307	0.064533904	-3.436585961	0.000589095	0.047333303	0.857508985
**LGALS1**	17001.20684	-0.213996969	0.051734363	-4.13645701	3.53E-05	0.006399761	0.862145356
**CSRP1**	9853.902813	-0.211507543	0.052043763	-4.0640325	4.82E-05	0.006738586	0.863634305
**PSMD2**	15841.83776	-0.207713041	0.057777594	-3.595044835	0.000324336	0.031709222	0.865908782
**KRT18**	6041.189078	-0.206499191	0.057664299	-3.58105784	0.000342206	0.032130766	0.866637644
**HSPD1**	10437.22276	-0.200860955	0.057785112	-3.475998383	0.000508955	0.044854314	0.870031201
**CAP1**	28038.28604	-0.188731379	0.046440295	-4.0639574	4.82E-05	0.006738586	0.877376896

Genes significantly upregulated in podocytes exposed to treatment *versus* remission serum

Upregulated in remission serum	baseMean	log2FoldChange	lfcSE	stat	pvalue	padj	FoldChange
**RNF144B**	4384.114594	0.244514647	0.060021927	4.073755374	4.63E-05	0.006738586	1.184694148
**IGFBP7**	10396.3523	0.245008493	0.07185342	3.409837612	0.000650016	0.047333303	1.185099748
**HSPB8**	2791.0892	0.245973093	0.07252816	3.39141503	0.000695327	0.047333303	1.185892382
**KIF5B**	2777.819963	0.268564834	0.071536519	3.754234036	0.000173872	0.018887689	1.204608909
**CD109**	1692.576842	0.301061631	0.078871921	3.817095176	0.000135032	0.016126052	1.232050704
**FAM84B**	3701.805495	0.30533803	0.087987162	3.470256604	0.000519961	0.044854314	1.235708136
**GTF2IP4**	3230.899303	0.319114188	0.08257187	3.864684064	0.000111233	0.014184636	1.247564312
**GTF2IP1**	1815.287714	0.490667694	0.102004876	4.810237619	1.51E-06	0.000631647	1.405095017

Journal	Year	n	Age	Diagnosis	Previous treatment	Vincristine regime	Steroid	Response
Paediatric Neph	1994	7	2-15 years	FSGS	Steroid resistant. CYC resistant 5	1.5mg/m^2 weekly/8 weeks	Prednisone 4 weeks: tapered	2 responded, 5 no response
Paediatric Neph	1998	8	1-14 years	6 FSGS, 1 familial FSGS, 1 mesangioproliferative GN	Prednisone and CYC in 8 (1: CyA added)	1.5mg/m^2 weekly/8 weeks	2 no. 6 unknown	2 CR, no response 5, 1 reduction on PCR
Paediatric Neph	2005	1	10 years	Kimura disease and NS	Prednisone			Remission (relapses responding to Rx)
Paediatric Neph	2005	12	6-14.5 years	SSNS/SDNS (no SRNS)	Prednisone, CYC, CyA in 10	1.5mg/m^2 weekly for 4 weeks then monthly for 4 months.	Vincristine alone in most	response 7, poor response 5. Trend to fewer relapses
Paediatric Neph	2006	17	1.2-14.4 years	9 SRNS (8 FSGS), 8 SDNS (3 FSGS, 5 MCD),	Prednisone, 12/17 CYC, 4/17 levamisole	1.5 mg/m^2 for 8 weeks	Unknown	3 CR
Biomed Res Int	2017	54	3.5-11.6 years	32 FSGS, 22 MCD/mesangial proliferation	Prednisone, CYC in all	1.5 mg/m^2 for 8 weeks	Prednisone tapered	21 CR, 7 PR, 26 no response<br>21 CR--- 6 relapsed in 1st 6 months<br>11, 9, 7 sustained CR at 12, 24, 60 months

Supplementary Table S3. Summary of studies administering vincristine in patients with glomerular diseases. FSGS, focal segmental glomerulosclerosis; NS, nephrotic syndrome; MCD, minimal change disease; CYC, cyclophosphamide; CyA, cyclosporine; CR, complete response; PCR, protein:creatinine ratio; PR, partial reduction.
